# Significance of risk polymorphisms for depression depends on stress exposure

**DOI:** 10.1038/s41598-018-22221-z

**Published:** 2018-03-02

**Authors:** Xenia Gonda, Gabor Hullam, Peter Antal, Nora Eszlari, Peter Petschner, Tomas GM Hökfelt, Ian Muir Anderson, John Francis William Deakin, Gabriella Juhasz, Gyorgy Bagdy

**Affiliations:** 1 Department of Psychiatry and Psychotherapy, Semmelweis University, Budapest, Hungary; 2 MTA-SE Neuropsychopharmacology and Neurochemistry Research Group, Hungarian Academy of Sciences, Semmelweis University, Budapest, Hungary; 3 Department of Pharmacodynamics, Faculty of Pharmacy, Semmelweis University, Budapest, Hungary; 4 Department of Measurement and Information Systems, Budapest University of Technology and Economics, Budapest, Hungary; 5 Retzius Laboratory, Department of Neuroscience, Karolinska Institutet, Stockholm, Sweden; 6 Neuroscience and Psychiatry Unit, Division of Neuroscience and Experimental Psychology, Faculty of Biology, Medicine and Health, University of Manchester, Manchester, United Kingdom; 7 Manchester Academic Health Sciences Centre, Manchester, United Kingdom; 8 Greater Manchester Mental Health NHS Foundation Trust, Prestwich, Manchester M25 3BL, United Kingdom; 9 SE-NAP 2 Genetic Brain Imaging Migraine Research Group, Semmelweis University, Budapest, Hungary; 10 NAP-2-SE New Antidepressant Target Research Group, Semmelweis University, Budapest, Hungary

## Abstract

Depression is a polygenic and multifactorial disorder where environmental effects exert a significant impact, yet most genetic studies do not consider the effect of stressors which may be one reason for the lack of replicable results in candidate gene studies, GWAS and between human studies and animal models. Relevance of functional polymorphisms in seven candidate genes previously implicated in animal and human studies on a depression-related phenotype given various recent stress exposure levels was assessed with Bayesian relevance analysis in 1682 subjects. This Bayesian analysis indicated a gene-environment interaction whose significance was also tested with a traditional multivariate analysis using general linear models. The investigated genetic factors were only relevant in the moderate and/or high stress exposure groups. Rank order of genes was *GALR2* > *BDNF* > *P2RX7* > *HTR1A* > *SLC6A4* > *CB1* > *HTR2A*, with strong relevance for the first four. Robust gene-gene-environment interaction was found between *BDNF* and *HTR1A*. Gene-environment interaction effect was confirmed, namely no main effect of genes, but a significant modulatory effect on environment-induced development of depression were found. Our data support the strong causative role of the environment modified by genetic factors, similar to animal models. Gene-environment interactions point to epigenetic factors associated with risk SNPs. Galanin-2 receptor, BDNF and X-type purin-7 receptor could be drug targets for new antidepressants.

## Introduction

Animal models of depression usually imply environmental factors, such as chronic unpredictable stress or learned helplessness. After such exposures behavioural phenotypes related to depression (e.g., anhedonia) and anxiety (e.g., increased fear, inhibited locomotor activity to discover new environment) develop in rodents. These animal models are widely used to study biological pathways involved in depression, to discover new target proteins for new antidepressants, to test potential new antidepressant compounds and to find candidate genes for depression. In turn, candidate genes were usually selected from data of stressed animals^1,2^, and evidence has also accumulated that depressogenic effects of different stressors are mediated by different biological pathways^3^.

This approach sharply contrasts genome-wide association studies (GWAS), where several thousands of patients (cases) and controls are included without any knowledge of presence or type of previous stressors^4^. GWAS are the state-of-the-art approach to test genetic main effects, but not interaction effects. In GWAS candidate genes generally do not show evidence for association with depression^5^ which led several researchers to question the candidate gene approach in general^6,7^. Furthermore, statistical power to detect interactions is typically less than for main effects^8^. In this study we selected seven candidate genes and used two completely different statistical approaches to test whether candidate gene approach has or has not relevance in a population with well-documented data on time and type of recent negative life events.

Family, twin, epidemiologic and molecular studies indicate that depression is a multifactorial illness showing a highly complex genetic architecture with a large number of loci, each contributing a very small effect size to the phenotype^5^. Heritability of depression depends on severity, estimated at approximately 37% in general population^9^, and 48–75% in hospital samples, the latter heritability of 75% shown in recurrent depression^10^. Specific gene variants have been difficult to identify^11^; positive findings in candidate gene studies have not been consistently replicated and very few statistically robust risk loci have emerged from genome-wide association studies (GWAS)^12^, although one recent study using the extremely large cohort with less intensive phenotyping approach identified 15 loci in major depression (Hyde *et al*. 2016). In contrast, environmental factors such as recent stressful life events and childhood trauma^13^ are associated with considerably greater risk of depression than specific genetic variants. Indeed, positive genetic studies of depression have emerged almost exclusively in interaction with adverse psychosocial exposures^11,14^.

As demonstrated by numerous studies the power to detect genetic influences may be increased by i) using “deep” or multiple related phenotypes, e.g. by considering a broader ‘multivariate’ phenotype than one categorical diagnosis^15^, ii) using risk groups, e.g. by separately analyzing those with marked exposure to psychosocial adversity^16^, and iii) using pathways, e.g. by using enrichment methods for aggregating the effect of variants toward gene and pathway levels^17^. We have previously demonstrated these by showing that functionally related gene variants within a pathway from genomically diverse regions show similar GxE interactions with respect to a complex phenotype^14,18^. The applied Bayesian multivariate methods allow these 3 approaches to be combined; they blur the distinction between dependent and independent variables and instead evaluate the strength of all, or a predefined set of possible relationships across variables. Using these methods, we have reported convergence of functionally related genes within the signaling pathway of the neuropeptide galanin on a multivariate phenotype, including quantitative measure of anxiety and depression, which operated only in those exposed to environmental stress. The use of the broader multivariate phenotype including symptoms of depression and also anxiety after stressful events also parallels animal models like chronic unpredictable stress.

In the present study we investigated whether candidate genes from both animal models and human studies across different pathways implicated in depression also converge to show a strong main effect and/or influence risk in the context of environmental stress. We quantified and compared the Bayesian relevance of these genes in populations differently exposed to stressful recent negative life events and also sought gene-gene interaction effects. Thus we selected candidate genes *a priori* based on: i) evidence of role in depression in animal models and also in our previous human studies, ii) having functional polymorphisms if possible, and iii) directly or indirectly influencing the main putative pathways of depression, including serotonin, HPA, neuropeptide, neurotrophin, endocannabinoid, and neuroinflammatory mechanisms. The following gene polymorphism were selected: rs6265 in *BDNF*^18^, rs8836 in *GALR2*^14^, rs7766029 in *CB1*^19^, rs6311 in *HTR2A*^20^, rs7958311 in *P2RX7*^21^, rs6295 in *HTR1A*^22^ and *5-HTTLPR* in *SLCA4*^16^.

## Results

The population was in Hardy-Weinberg equilibrium (p-value > 0.05). Description of the population is shown in Table 1. The three elements of the multiple phenotype showed strong correlations with each other confirming the relevance of the multiple depression related phenotype (Table S1).

**Table 1:** Population description.

Demographics		ALL	BUD	MAN
gender	male (%)	509 (30.3%)	226 (33.7%)	283 (28%)
	female (%)	1173 (69.7%)	445 (66.3%)	728 (72%)
age	mean (SD)	33.23 (10.53)	31.78 (10.54)	34.2 (10.42)
lifetime depression (DEP)	no (%)	958 (57%)	524 (78.1%)	434 (42.9%)
	yes (%)	724 (43%)	147 (21.9%)	577 (57.1%)
recent negative life events (RLE)				
	low (%)	1134 (67.4%)	479 (71.8%)	651 (64.4%)
	moderate (%)	311 (18.5%)	120 (18%)	191 (18.9%)
	high (%)	237 (14.1%)	68 (10.2%)	169 (16.7%)
current depression score (BSI-DEP)				
	low (%)	1133 (67.4%)	543 (80.9%)	590 (58.4%)
	moderate (%)	290 (17.2%)	92 (13.7%)	198 (19.6%)
	severe (%)	259 (15.4%)	36 (5.4%)	223 (22.1%)
current anxiety score (BSI-ANX)				
	low (%)	1096 (65.2%)	503 (75%)	593 (58.7%)
	moderate (%)	339 (20.2%)	122 (18.2%)	217 (21.5%)
	severe (%)	247 (14.7%)	46 (6.9%)	201 (19.9%)
**Genetic variables**				
*5-HTTLPR*	ss (%)	307 (18.3%)	114 (17%)	193 (19.1%)
	sl (%)	819 (48.7%)	328 (48.9%)	491 (48.6%)
	ll (%)	556 (33.1%)	229 (34.1%)	327 (32.3%)
rs6265 (*BDNF*)	GG (%)	1128 (67.1%)	442 (65.9%)	686 (67.9%)
	AG (%)	484 (28.8%)	202 (30.1%)	282 (27.9%)
	AA (%)	70 (4.2%)	27 (4%)	43 (4.3%)
rs6295 (*HTR1A*)	GG (%)	425 (25.3%)	174 (25.9%)	251 (24.8%)
	GC (%)	845 (50.2%)	366 (54.5%)	479 (47.4%)
	CC (%)	412 (24.6%)	131 (19.5%)	281 (27.8%)
rs6311 (*HTR2A*)	CC (%)	569 (33.8%)	224 (33.4%)	345 (34.1%)
	CT (%)	832 (49.5%)	314 (46.8%)	518 (51.2%)
	TT (%)	281 (16.7%)	133 (19.8%)	148 (14.6%)
rs7766029 (*CB1*)	CC (%)	443 (26.3%)	164 (24.4%)	207 (20.5%)
	CT (%)	868 (51.6%)	352 (52.5%)	516 (51%)
	TT (%)	371 (22.1%)	155 (23.1%)	288 (28.5%)
rs7958311 (*P2RX7*)	GG (%)	983 (58.4%)	422 (62.9%)	561 (55.5%)
	AG (%)	610 (36.3%)	212 (31.6%)	398 (39.4%)
	AA (%)	89 (6.3%)	37 (5.5%)	52 (5.1%)
rs8836 (*GALR2*)	CC (%)	580 (34.5%)	216 (32.2%)	364 (36%)
	CG (%)	820 (48.7%)	338 (50.4%)	482 (47.7%)
	GG (%)	282 (16.8%)	117 (17.4%)	165 (16.3%)

SD: standard deviation; BSI: Brief Symptom Inventory.

**Figure 1:**
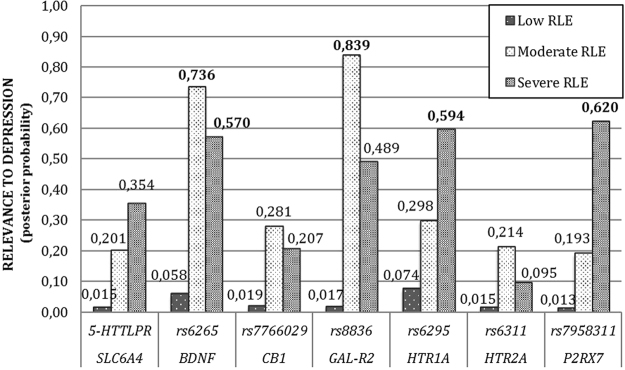
Relevance of genetic factors with respect to multiple depression-related phenotype in persons with low, moderate or high exposure to recent negative life events (RLE). Possible dependency relationship models of genetic variables were investigated for each RLE subgroup. Then, relying on Bayesian model averaging an individual posterior probability score was computed for each genetic factor. Results indicate that these genetic factors are relevant to depression only in subjects with moderate or severe RLE exposure. In addition, several factors such as *BDNF*, *GAL-R2*, *HTR1A*, and *P2RX7* appear more relevant with respect to depression in moderate and severe RLE subgroups than the highly investigated serotonin transporter related *5-HTTLPR*. Posterior probabilities of >0.50 indicate strong relevance and are marked with bold numbers.

Bayesian relevance analysis revealed that the investigated genetic factors were only relevant to the multiple depression-related phenotype in case of moderate or high RLE exposure (Fig. 1**)**. In case of the low RLE exposure group all genetic factors were non-relevant with low posterior probabilities (pr <0.1). In this analysis relevance increases in parallel with posterior probability, and a strong relevance is associated with pr >0.5. Moderate and/or high RLE exposure levels entailed an increased relevance of all SNPs compared to the low exposure case. For the investigated SNPs some were more relevant in the moderate, some others in the high RLE exposure groups. On one hand, SNPs rs6265 (*BDNF*) and rs8836 (*GALR2*) were strongly relevant with high posterior probabilities (pr = 0.74 and pr = 0.84 respectively) after moderate exposure. On the other hand, rs7958311 (*P2RX7*), rs6295 (*HTR1A*) and *5-HTTLPR* (*SLC6A4*) appeared more relevant in the high RLE exposure group. Among those the rs7958311 (*P2RX7*) SNP was the most relevant (pr = 0.62) followed by rs6295 (*HTR1A*) (pr = 0.59) and rs6265 (*BDNF*) (pr = 0.57). Comparatively, *5-HTTLPR* (*SLC6A4*), rs7766029 (*CB1*) and rs6311 (*HTR2A*) had relatively low posteriors even after moderate or high exposure, and can be considered less relevant in this multivariate model.

**Figure 2:**
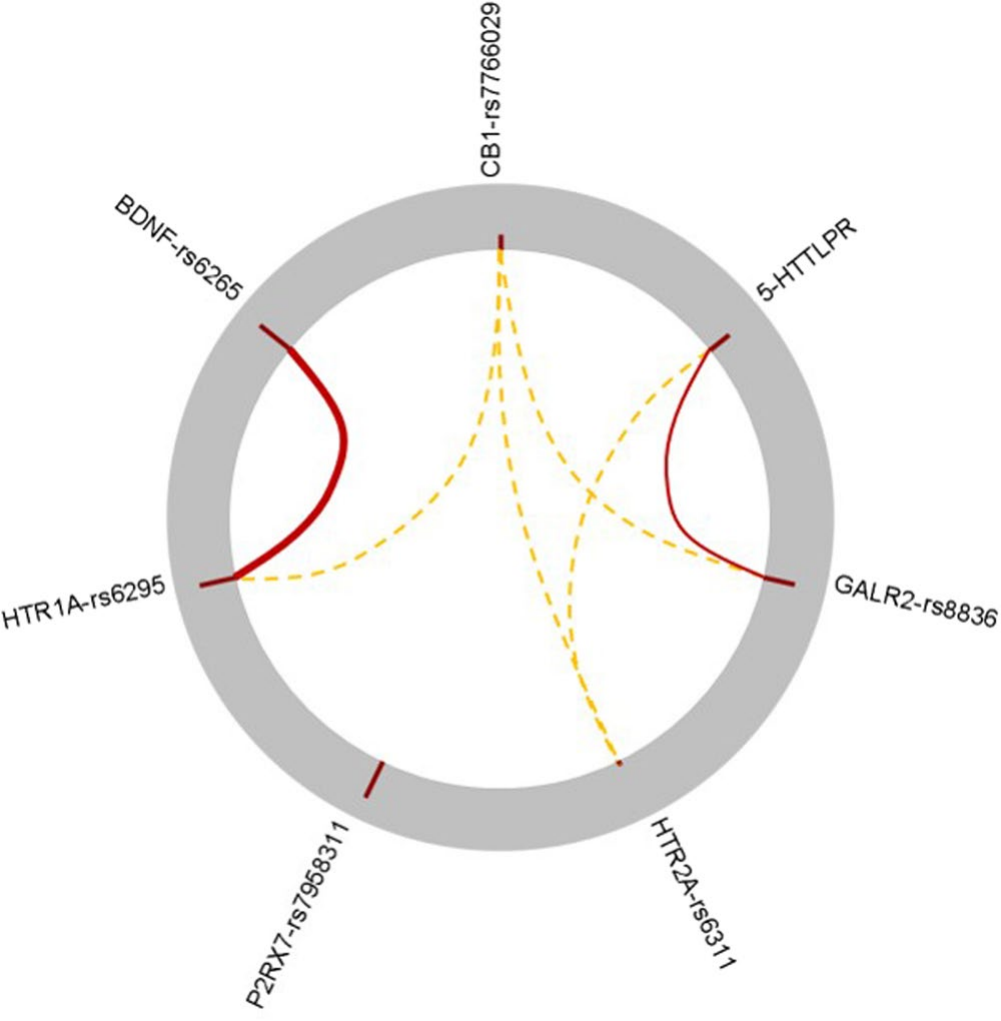
Statistical interactions between genetic factors with respect to multiple depression-related phenotypes in persons with high exposure to recent negative life events. A Bayesian interaction score was computed for all possible variable pairs in order to measure their joint occurrence in relevant models with respect to multiple depression-related phenotypes. The individual relevance of each SNP is shown in the outer grey ring with red columns having a height proportional to their relevance. Possible interactions between SNPs are represented by curved red lines between corresponding columns with a width proportional to the likeliness of the interaction. Interactions related to SNPs with low individual relevance (e.g. *CB1* and *HTR2A* SNPs, denoted with dashed orange lines) can be neglected, as the interaction score is sufficient only in case of variables with moderate or high relevance. Results indicated two interactions: a strong interaction between rs6265 (*BDNF*) - rs6295 (*HTR1A*) and a mild interaction between *5-HTTLPR* (*SLC6A4*) – rs8836 (*GALR2*). The rs6265 (*BDNF*) - rs6295 (*HTR1A*) interaction was also observed in the GLM analysis of models (see Table 2). In contrast, the interaction of *5-HTTLPR* (*SLC6A4*) – rs8836 (*GALR2*) was not revealed by the GLM analysis, which indicates that this interaction is of a different type, and only detectable by systems-based methods. Note that results shown in Fig. 1 suggest that the *5-HTTLPR* (*SLC6A4*) and rs8836 (*GALR2*) SNPs have separate main effects with respect to depression.

Two gene-gene interactions were found in the data (Fig. 2). A strong interaction between rs6265 (*BDNF*) and rs6295 (*HTR1A*) was revealed by both Bayesian relevance analysis and the GLM analysis of the models (Table 2). Another weak interaction between *5-HTTLPR* (*SLC6A4*)-rs8836 (*GALR2*) was also suggested by Bayesian relevance analysis, but was not confirmed by the GLM analysis. It is noteworthy that the P2RX7 polymorphism clearly stands alone, its effect is completely apart from any other SNPs included in the analysis, as shown on Fig. 2.

**Table 2:** Comparison of GLM models indicating the importance of gene-environment interactions. Three GLM models were constructed (see Fig. 1 for an illustration of included dependency relationships): (1) Model-1 containing only main effect of the environmental factor (relationship ‘a’), and (2) Model-2 containing only main effects of the environmental and genetic factors (‘a’ + ‘b’), and (3) Model-3 containing main effects plus interactions of the environmental and genetic factors (‘a’ + ‘b’ + ‘c’). Note that the inclusion of gene-environment interaction in Model-3 provides a significantly better model compared to Model-2 containing only main effects.

Compared Models		DF-1	DF-2	F	p-value	Variance explained	
**Model 1 (‘a’)** RLE	**Model 2 (‘a’ + ‘b’)** RLE + genetic factors (only main effects)	4	18	1.55	0.086	**Model 1** 6.13%	**Model 2** 7.03%
**Model 1(‘a’)** RLE	**Model 3 (‘a’** + **‘b’** + **‘c’)** RLE + genetic factors (main effects + interactions)	4	38	1.88	**0.002**	**Model 1** 6.13%	**Model 2** 8.68%

DF-1 and DF-2 represents degrees of freedom for compared models, and F represents the value of the corresponding F statistic.

**Figure 3:**
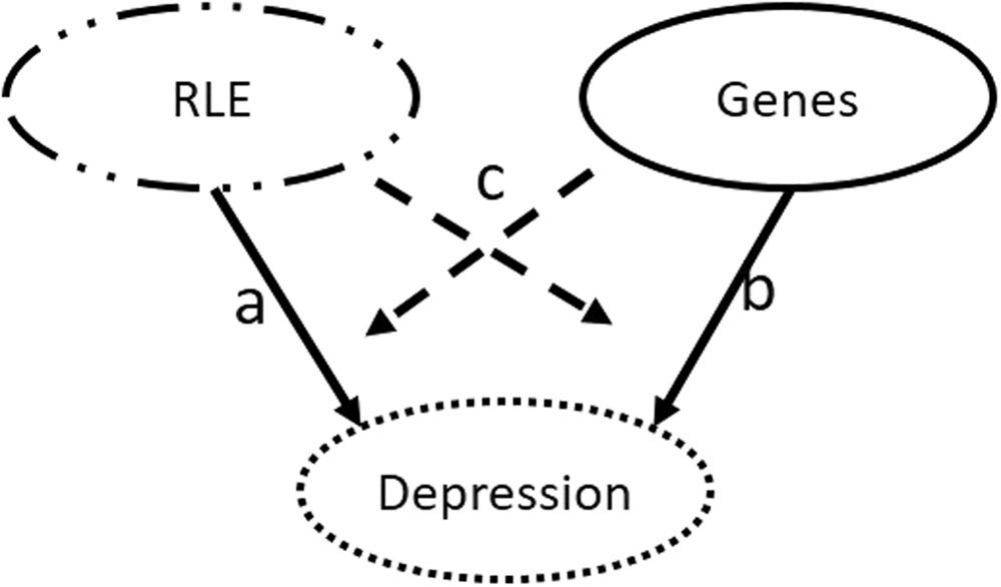
Illustration of dependency relationships included in GLM models investigating the role of genetic and environmental factors with respect to depression phenotypes. Relationships ‘**a**’ recent life events (RLE) and ‘**b**’ represent the main effect of genetic factors, respectively, on depression phenotypes. Relationship ‘**c**’ represents the interaction between genetic factors and RLE. Model 1: effect of ‘**a**’. Model 2: effect of ‘**a** + **b**’. Model 3: effect of ‘**a** + **b** + **c**’.

The Bayesian relevance analysis indicated a strong gene-environment interaction between the investigated genetic factors and RLE exposure. In order to confirm this interaction, a frequentist analysis involving GLM was performed using depression phenotypes as dependent variables, and age and gender as cofactors. Model-1 served as the basis of comparison containing only RLE. Both Model-2 and Model-3 were compared to Model-1, for further explanation see Fig. 3. Note that Model-1 was significantly different (due to RLE) from a null model consisting of only age and gender (p-value < 10^−5^). Model-2 containing only main effects of RLE and genetic factors is not significantly different (in terms of residual variance) from Model-1 containing only RLE (F = 1.55, p-value = 0.086). However, Model-3 including also the interaction between genetic factors and RLE is significantly different compared to Model-1 (F = 1.887, p-value = 0.0016). In addition, an analysis of explained variance of the depression phenotype was also performed. Model-1 (relationship ‘a’, Fig. 3) explains 6.13% of variance, in contrast with a model containing only main effects of genetic factors corresponding to relationship ‘b’ which explains 2.91% of variance. Model-2 (‘a’ + ‘b’), however, in terms of explained variance (7.03%) is not significantly different from Model-1 (Table 2). On the other hand, the interactions between RLE and genetic factors (relationship ‘c’, Fig. 3) explain a considerable amount of variance, making Model-3 (‘a’ + ‘b’ + ‘c’, 8.68%) significantly different from Model-1. Table 2 presents the comparison of the previously described three models all of which included age and sex as covariates.

**Figure 4:**
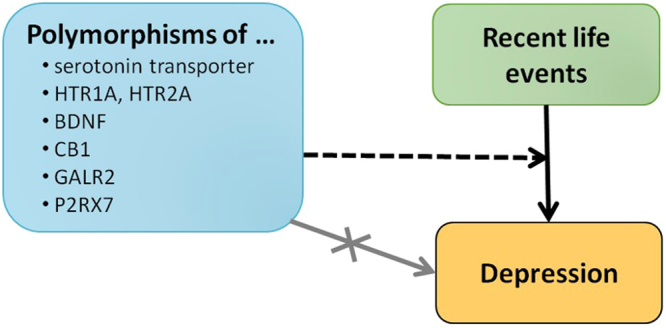
The role of all investigated candidate genes reveals in gene-environment interactions instead of main effects in the development of depression. Results of this study indicate that the polymorphisms of the investigated candidate genes have negligible main effects on depression. Instead, the interactions of these genetic variables in interaction with environmental factors, such as exposure to stress influence the development of depression.

In short, these results show that effects of investigated genetic factors can be decomposed to non-significant main effects and significant gene-environment interactions (Fig. 4).

A logistic regression analysis with age, gender and population as covariates indicated no main effects of the polymorphisms on depression (data not shown).

Finally, possible biases resulting from population differences and RLE exposure categories were investigated. A Bayesian analysis involving a population descriptor variable was carried out on the whole dataset as well on all RLE subpopulations. The analysis involving the whole dataset indicated no direct dependencies with respect to the population descriptor (pr <0.07), suggesting that there is no population stratification bias in this sample regarding the investigated SNPs. In case of the RLE subpopulations, inclusion of the population descriptor caused only minor differences (<0.15) in the relevance posteriors of genetic variables supporting stable genetic effects (Supplementary Figure S1). Similarly, no direct dependencies between RLE and genetic variables were detected (pr <0.02), thus the gene-environment interaction with respect to depression phenotypes are not driven by direct RLE-gene associations. In addition, GLM models using also a continuous RLE variable were investigated. The difference between the previously described Model-3 and the Model-1 remained significant (F = 1.567, p-value < 0.05) indicating a robust effect of RLE independently of scaling.

## Discussion

Our study showed that the contribution of multiple previously identified risk functional polymorphisms of candidate genes mapping several important neurobiological pathways, implicated in depression and also showing stress-dependent effects in previous animal studies, was only manifested in persons exposed to at least moderately to recent life events. They did not converge to show a detectable main effect, but together they significantly influenced the risk in the context of environmental stress. This was confirmed by Bayesian multivariate methods, namely, no relevance of any of the investigated risk variants was detected in persons with low exposure. For the moderately exposed population only the galanin-2 receptor and BDNF polymorphisms were relevant. The rank order of the genes was *GALR2* > *BDNF* > *P2RX7* > *HTR1A* > *SLC6A4* > *CB1* > *HTR2A*, with high relevance for the first four, among all exposure groups. Furthermore, 5-HTTLPR, the most extensively investigated polymorphism with respect to interaction with life events^16,23,24^ showed only very low relevance (Fig. 3). Since the functional effect, in other words its effect on mRNA expression, of this genetic variant is the strongest among all of these on a molecular level, these data suggest that several other genes play a more prominent role in mediating the effects of recent stressors on depression. Our results also reflect that effect of risk variants may be activated differentially depending on the level of exposure to stressors, and that the effects of differing severity of stress exposure may be mediated by partially different pathways and mechanisms.

Our study applied a multivariate depression-related phenotype incorporating lifetime depression, current depression, and anxiety to encompass various aspects of depression including also quantitative traits (e.g., depression and anxiety symptoms). This approach may have power advantage over diagnostic categories in epidemiological, population genetic samples^14,25,26^. Defining phenotype is crucial in the case of depression which is a highly heterogeneous phenomenon^27^. There appears to be a significant overlap between depression and anxiety phenotypes, expressed by the diagnostic category of anxious depression, and there is also high comorbidity between the two, with a significant impact on both the course and treatment of these disorders^28^. Besides both being stress-related disorders, there is also a significant proportion of shared genetic risk^28^, with several polymorphisms implicated in both depression and anxiety, arguing for a complex approach to this phenotype. Furthermore, the validity of self-report phenotype for major depression has been shown in a GWAS recently^29^. The phenotype including symptoms of depression and also anxiety after stressful events also parallels animal models like chronic unpredictable stress^30,31^. The independence of the used phenotypes would argue for their individual testing, but the strong and incomplete correlation among the elements also confirms the use of our multiple phenotype.

Another important novel aspect of our study is considering several functional polymorphisms previously described in human and animal studies from distinct pathways including the serotonergic and endocannabinoid systems, neuropeptides, neuroinflammation and neuroplasticity in one multivariate model. In our study we targeted depression as a complex disease and a product of multiple networks, where the activity of the pathways may be shaped by environmental experience^32^, which was quantified in each person.

Compared to heritability which accounts for 37–42% in the variance in general population samples^9^, influence of environmental effects is estimated at 63% in depression^28^. Etiologically relevant distal and proximal stressors are relatively common, and while frequency of severe life events is estimated to be one in every 3–4 years, depression is triggered in only about one fifth of those with acute stress exposure^33^. There is a pronounced individual variation in the sensitivity towards environmental exposure at least in part due to genetic factors, and several genes are not directly involved in the development of depression but via influencing vulnerability towards effects of environmental stressors^10,12^.

The relationship between environmental and genetic influences is manifold. The effect of genetic and environmental factors may be additive, or genetic variants may increase the likelihood of subjects seeking risk environments or life events causing gene-environment correlation as shown by twin and adoption studies^34–36^. The exact nature of the interaction between genes and environment (GxE) is controversial. While the diathesis-stress model^37^ proposes that vulnerability towards environmental triggers depends on the biological background, including the genotype, which buffers or exacerbates the effect of stressors, the differential susceptibility model^38^ incorporates a possible positive environmental aspect^5^, postulating that the biological context modulates the sensitivity to both positive and negative environmental influences, making individuals not vulnerable, but ‘plastic’, in their response to the environment^39^.

Lack of consideration of such environmental effects may be one prominent reason for the failure of replication in candidate gene studies and GWAS into the genetic background of depression^25,40^. The only GWAS where additional genome-wide by environment interaction study (GWEIS) was also performed included population data from African American and Hispanic/Latina women^25^. Despite the fact that the population was markedly different from ours, including ethnicity, gender, age, prevalence of negative life events and stress exposure, there are important conclusions from that study. No genome-wide significant main effect was found in the GWAS, but a significant GWEIS hit was described in the African American sample. Furthermore, depressive symptoms and stressful life events were strongly genetically correlated^25^. Interaction of polygenic risk scores with environmental effects has been investigated in a similarly small number of studies, with negative results for interaction with recent life events^11,13^.

Our results throw light on a further important aspect of GxE in the development of depression, that is, whether environmental factors play a permissive role modulating the manifestation of genetically-based phenotypes or a causative role, with genetic factors only modifying this effect. It has already been proposed that genes act by modifying vulnerability towards environmental influences rather than directly playing a causative role in the development of depression^10,12^. This may be even more evident in our candidate gene selection, as animal models of depression usually depend on stress-induced depression-like behaviour. The larger increase in depression risk due to an interaction between genetic effects and life events than expected by combining their individual effects (Fig. 3 and Table 2) is an important argument against the first assumption, see also^11,14^, and thus, our results suggest that risk genetic variants may show differential activation as a function of evironmental exposure. Furthermore, effects caused by different severity of exposure may be mediated by partially different pathways. These data indicate a causative role for the environment, only modified by the genetic factors studied here. In other words, our results show that genes described here do not have a weak main effect, but rather a detectable modulatory effect.

Our study has several limitations. Our multiple depression-related phenotype and recent life events were measured based on self-report and these are subject to recall and reporting bias, although we applied only validated instruments and validated our phenotype in a subsample, furthermore the validity of self-report phenotype for major depression has been validated also in a GWAS recently. In addition, lifetime depression (also included in our multiple phenotype) was not controlled for timing of recent life events. Furthermore, this is a hypothesis driven candidate gene study which assessed a limited number of genetic variants (functional polymorphisms in seven candidate genes previously implicated in animal and human depression studies) on a moderately large sample. Therefore, results can be potentially inflated, and an independent study is required in the future to validate these results preferably using imputed GWAS data. That would allow the investigation of the contributions of all genes in the genome in the context of environmental stressors. The application of Bayesian systems-based methods would be advantageous in such a scenario as well, as they are better powered to uncover modest interaction effectsthan non-Bayesian methods using correction against multiple hypothesis testing. However, the computational scaling of systems-based methods to a genome-wide level still requires further research.

With these limitations in mind, our results suggest a potential role of the candidate gene-environment interaction. They indicate a complex interaction pattern between candidate genetic variants in different neurobiological pathways and severity of exposure to life events in the emergence of depression. Neither the separate, nor the combined effect of the investigated polymorphisms was detectable on depression in the absence of stress exposure. In contrast, several genes modulated the effects of recent stressors, and their effects converged to a significant modulation on the development of depression. Furthermore, different levels of stress exposure were modulated differently by distinct genes reflecting multiple mechanisms underlying the effects of different severity of life events in the development of depression. Given the near complete lack of effect of our studied polymorphisms in the absence of at least moderate exposure to recent stressors, it is obvious that effects of important genetic variants are overlooked in samples, if the presence of stressors has not been considered in depression. Also, it is possible that different pathways play a role in the development of depression depending on whether life events are present or not; and different types or even severity of life events may be modulated by different neural mechanisms and pathways. These data emphasize the need for incorporating environmental variables in GWAS.

## Methods and Materials

### Study population

The study was part of the EU-funded NewMood study (New Molecules in Mood Disorders, Sixth Framework Program of the EU, LSHM-CT-2004-503474) approved by local Ethics Committees (North Manchester Local Research Ethics Committee, Manchester, UK; Scientific and Research Ethics Committee of the Medical Research Council, Budapest, Hungary), and carried out in accordance with the Declaration of Helsinki and all relevant guidelines and regulations. Before participating, all subjects provided written informed consent.

Description of the population and questionnaires have been published previously^14,18,19^. In this current study we investigated how genetic variants of candidate genes interact with life-stressors on a multiple depression-related phenotype in a European population cohort of 1682 subjects which is a subset of the NewMood study population consisting of subjects that were successfully genotyped with respect to all seven investigated genetic factors and had available data for all parameters included in this analysis. Recent negative life events (RLE), lifetime depression, Brief Symptom Inventory (BSI) depression and anxiety scores were determined in each subject. Relevant population data are provided in Table 1.

### Phenotype

In the present study, we analyzed reported lifetime depression, current depression and anxiety jointly as a multiple depression-related phenotype described previously^14,24^. Reported lifetime depression (DEP) was determined using the background questionnaire. Subsequently, the Structured Clinical Interview for DSM-IV (SCID) was used to validate findings^18^. Current depressive symptoms were measured based on the depression items plus the additional items (BSI-DEP), and anxiety using the anxiety items (BSI-ANX) of the BSI^41^. A weighted score was computed separately for BSI-DEP and BSI-ANX variables by summing the corresponding item scores divided by the number of items completed and categorized into low (0 − < 1), moderate (1 − < 2), and severe (2–4) categories.

In order to identify recent negative life events (RLE) we used the List of Threatening Experiences questionnaire^42^ which queried problems related to illnesses/injuries, financial difficulties, problems related to intimate relationships, and social network occurring in the last year. Based on corresponding items the number of RLEs was counted for each subject, and categorized (low = 0–1, moderate = 2, high = 3/more) as used in our previous studies^19^. The resulting low, moderate and high RLE exposure groups were used in the statistical analysis.

### Genotyping

We genotyped seven a priori selected polymorphisms (rs6265, rs8836, rs7766029, rs6311, rs7958311, rs6295 and *5-HTTLPR*) based on evidence from previous studies on involvement in depression. Buccal mucosa cells were collected for genotyping using a cytology brush (Cytobrush plus C0012, Durbin PLC, Harrow UK) and 2.0 mL collection buffer in 15-mL plastic tubes. Genomic DNA was extracted according to a previously published protocol^43^. Laboratory work was carried out according to the ISO 9001:2000 quality management requirements and was blinded with regard to phenotype.

### Statistical methods

In order to efficiently explore relevant factors and interactions for complex phenotypes represented by multiple variables, we applied the Bayesian network based Bayesian multi-level analysis of relevance BN-BMLA (Antal *et al*., 2008), which is referred to as Bayesian relevance analysis in the paper. This method utilizes special hierarchical structural properties of Bayesian networks representing essential concepts in relevance analysis such as Markov blanket memberships, Markov blanket sets, and Markov blanket graphs. Bayesian relevance analysis applies a Markov Chain Monte Carlo sampling method to perform a random walk in the space of directed acyclic graphs^44^. This MCMC process results in samples of DAG structures which are used to estimate the consistent posteriors for structural properties representing relevance, e.g. posteriors for Markov blanket memberships representing strong relevance^45^. Thus this method quantifies the relevance of variables with respect to one or more selected target variables as probability scores (posterior probability of strong relevance) relying on Bayesian model averaging^46^.

In this study, all genetic factors were analyzed jointly in a multivariate model^14,24^, specifically we aimed to investigate the relevant relationships concerning the depression related complex phenotype described by three variables (DEP, BSI-DEP, BSI-ANX) and possible statistical interactions with recent negative life events. The Markov blanket graphs representing these relations are the hypotheses in our approach, which takes into account the potential interdependencies of the target variables. Because the Bayesian approach induces a posterior distribution over this hypothesis space, it provides an efficient handling of multiple hypothesis testing.

The Bayesian relevance analysis was configured and executed to estimate Markov blanket membership posterior probabilities using a uniform structure prior, a Cooper-Herskovits parameter prior^47^ with 10^6^ burn-in and 5 × 10^6^ normal steps. In addition, the maximum number of parents (i.e. number of incoming edges) was limited to 5. The parameter prior was non-informative with respect to the data set, and it was selected based on our previous experiments with parameter priors^48^. Analyses were carried out for each RLE exposure group separately. Age and gender was included in each multivariate model. Note that the multivariate model provided by Bayesian relevance analysis allows the investigation of interactions on a system level, which is in line with recent recommendations regarding GxE analyses^7^.

In addition, a frequentist multivariate analysis using general linear models (GLM) was applied to test the significance of the interaction of the investigated genetic factors and recent negative life events versus the effect of recent negative life events on the multiple depression-related phenotype. Three GLM models were constructed (Fig. 3, Table 2**)** for an illustration of included dependency relationships): (1) Model-1 containing only main effect of the environmental factor (relationship ‘a’), and (2) Model-2 containing only main effects of the environmental and genetic factors (‘a’ + ‘b’), and (3) Model-3 containing main effects of the environmental and genetic factors (see Model-2) plus interactions of environmental and genetic factors (‘a’ + ‘b’ + ‘c’). Age and gender were included as cofactors in all models. The residual variance of the models was compared and tested using an F-statistic. GLM analyses were performed with SPSS21.0 for Windows (IBM, Armonk, NY) using a significance threshold 0.05.

### Data availability

The datasets generated and analysed during the current study are available in the Figshare repository, https://figshare.com/s/737f45fe41522e601d0a.

## Electronic supplementary material

Supplementary Information
