# Supplementary material for: Significance of risk polymorphisms for depression depends on stress exposure
Source: Sci Rep. 2018 Mar 2;8:3946. doi: 10.1038/s41598-018-22221-z (PMC5834495; doi:10.1038/s41598-018-22221-z)

Supplementary Information

Significance of risk polymorphisms for depression depends on stress exposure

Xenia Gonda, Gabor Hullam, Peter Antal, Nora Eszlari, Peter Petschner, Tomas GM Hökfelt, Ian Muir Anderson, John Francis William Deakin, Gabriella Juhasz, Gyorgy Bagdy

Supplementary Figure S1. Differences in the two populations (Budapest and Manchester) are not responsible for the marked differences in the relevance of the SNPs in either the moderately or highly exposed population

The effect of population differences was investigated by including a population descriptor variable (indicating the origin of the sample: Budapest or Manchester) in Bayesian multivariate models for moderate and high RLE exposure groups. The resulting posterior probabilities, that is the relevance of genetic variables with respect to depression-related phenotypes (RLE-H-POP: high RLE exposure, RLE-M-POP: moderate RLE exposure) were compared with those of the corresponding original model (RLE-H and RLE-M). Results indicate only minor changes in relevance posteriors for both the moderately and the highly exposed subpopulations.

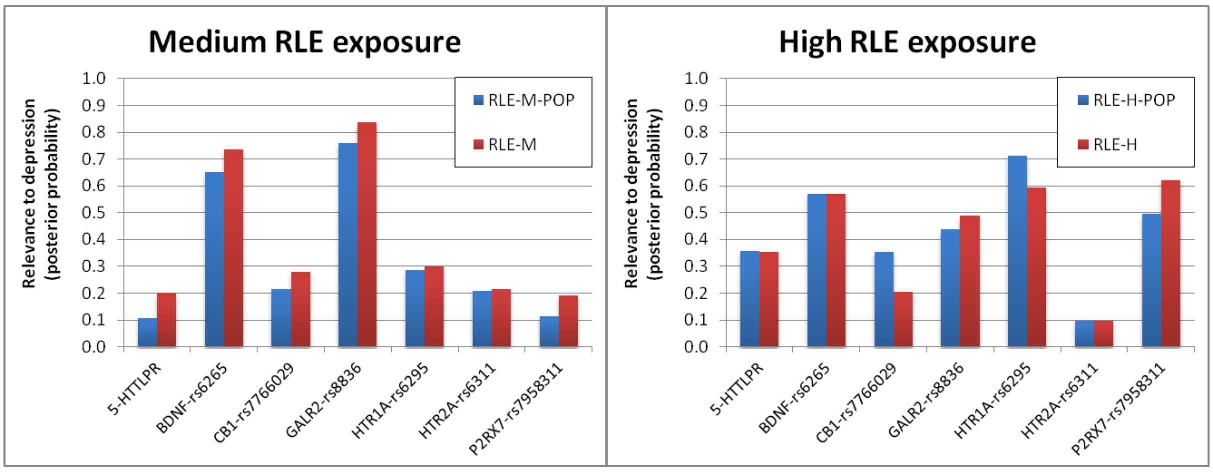

Supplement: Supplementary file 1 — Supplementary Information [file 41598_2018_22221_MOESM1_ESM.pdf]
